# Development and validation of a machine learning model to predict the risk of lymph node metastasis in renal carcinoma

**DOI:** 10.3389/fendo.2022.1054358

**Published:** 2022-11-18

**Authors:** Xiaowei Feng, Tao Hong, Wencai Liu, Chan Xu, Wanying Li, Bing Yang, Yang Song, Ting Li, Wenle Li, Hui Zhou, Chengliang Yin

**Affiliations:** ^1^ Department of Neuro Rehabilitation, Shaanxi Provincial Rehabilitation Hospital, Xi ‘an, China; ^2^ Department of Cardiac Surgery, Fuwai Hospital Chinese Academy of Medical Sciences, Shenzhen, Shenzhen, China; ^3^ Department of Orthopaedic Surgery, the First Affiliated Hospital of Nanchang University, Nanchang, China; ^4^ Department of Clinical Medical Research Center, Xianyang Central Hospital, Xianyang, China; ^5^ Life Science Department, Tianjin Prosel Biological Technology Co., Ltd, Tianjin, China; ^6^ Department of Gastroenterology and Hepatology, Chinese People's Liberation Army (PLA) General Hospital, Beijing, China; ^7^ Department of Cell Biology, College of Basic Medical Sciences, Tianjin Medical University, Tianjin, China; ^8^ State Key Laboratory of Molecular Vaccinology and Molecular Diagnostics & Center for Molecular Imaging and Translational Medicine, School of Public Health, Xiamen University, Fujian, China; ^9^ School of Pharmacy, Tianjin Medical University, Tianjin, China; ^10^ Faculty of Medicine, Macau University of Science and Technology, Macau, Macau SAR China

**Keywords:** kidney cancer, renal cell cancer, lymph node metastasis, machine learning, predictive model, web calculator

## Abstract

**Simple summary:**

Studies have shown that about 30% of kidney cancer patients will have metastasis, and lymph node metastasis (LNM) may be related to a poor prognosis. Our retrospective study aims to provide a reliable machine learning-based model to predict the occurrence of LNM in kidney cancer. We screened the pathological grade, liver metastasis, M staging, primary site, T staging, and tumor size from the training group (n=39016) formed by the SEER database and the validation group (n=771) formed by the medical center. Independent predictors of LNM in cancer patients. Using six different algorithms to build a prediction model, it is found that the prediction performance of the XGB model in the training group and the validation group is significantly better than any other machine learning model. The results show that prediction tools based on machine learning can accurately predict the probability of LNM in patients with kidney cancer and have satisfactory clinical application prospects.

**Background:**

Lymph node metastasis (LNM) is associated with the prognosis of patients with kidney cancer. This study aimed to provide reliable machine learning-based (ML-based) models to predict the probability of LNM in kidney cancer.

**Methods:**

Data on patients diagnosed with kidney cancer were extracted from the Surveillance, Epidemiology and Outcomes (SEER) database from 2010 to 2017, and variables were filtered by least absolute shrinkage and selection operator (LASSO), univariate and multivariate logistic regression analyses. Statistically significant risk factors were used to build predictive models. We used 10-fold cross-validation in the validation of the model. The area under the receiver operating characteristic curve (AUC) was used to assess the performance of the model. Correlation heat maps were used to investigate the correlation of features using permutation analysis to assess the importance of predictors. Probability density functions (PDFs) and clinical utility curves (CUCs) were used to determine clinical utility thresholds.

**Results:**

The training cohort of this study included 39,016 patients, and the validation cohort included 771 patients. In the two cohorts, 2544 (6.5%) and 66 (8.1%) patients had LNM, respectively. Pathological grade, liver metastasis, M stage, primary site, T stage, and tumor size were independent predictive factors of LNM. In both model validation, the XGB model significantly outperformed any of the machine learning models with an AUC value of 0.916.A web calculator (https://share.streamlit.io/liuwencai4/renal_lnm/main/renal_lnm.py) were built based on the XGB model. Based on the PDF and CUC, we suggested 54.6% as a threshold probability for guiding the diagnosis of LNM, which could distinguish about 89% of LNM patients.

**Conclusions:**

The predictive tool based on machine learning can precisely indicate the probability of LNM in kidney cancer patients and has a satisfying application prospect in clinical practice.

## Introduction

Kidney cancer is among the 10 most commonly diagnosed malignant tumors in the United States (1). As a serious public health problem, kidney cancer annually contributes to more than 400,000 diagnosed new cases and 175,000 victims worldwide (2). Renal cell carcinoma (RCC) composed 90% of primary malignant kidney tumor cases (1, 3). About 30% of RCC patients present with metastases (1), and 15% have relapses in distant sites (4, 5) since various mutated genes and histology. The lymph node is one of the most frequent metastatic sites of RCC (4, 6)and may associate with a poor prognosis (7–11). The 5-year survival rate of localized RCC is near 93%. But for those with distant metastatic, the survival rate decreases to 12% (12, 13).

Although great progress had been made in treatments for RCC metastatic, such as immunosuppressants and targeted drugs, these could not stop RCC from relapsing, and relapse is still a big challenge for mankind (14). Early detection could greatly ameliorate the survival rate (15). Predicting lymph node status in RCC patients needs immediate attention by the development of new diagnostic tools.

Great efforts had been paid in developing predictive methods to identify LNM risk factors (16–21). As a novel and popular artificial intelligence tool, ML plays a vital role in improving predictive accuracy in diagnosis and prognosis (22, 23) and has been widely used in medical data analysis (24–26). Compared with other statistical methods, ML algorithms can allow interactions between variables, recognize potentially important predictor variables, find optimized algorithms between the outcome of interest and potential predictor variables by learning from dataset patterns, and have demonstrated greater accuracy in clinical settings (23, 27). Unfortunately, there are still no reports evaluating the LNM risk in RCC patients with ML algorithms.

Hence, we developed a brand-new predictive model with ML algorithms. This will help clinicians with individualized clinical decisions. To establish models, we extracted patient data from the Surveillance, Epidemiology, and End Results (SEER) databases and verified it with an independent external validation dataset.

## Materials and methods

### Data source and processing

The current study used SEER * Stat software (8.3.5) to extract patients diagnosed with kidney cancer cases from January 1, 2010, to December 31, 2017, as the training cohort.

Patients were enrolled as the following inclusion criteria: (1) The participants were identified with primary kidney cancer identified by using universal morphology codes (8120/3, 8130/3, 8260/3, 8310/3, 8312/3, 8317/3) according to the International Classification of Diseases for Oncology codes (3rd edition); (2) Complete demographic and clinical data including demographic characteristics (marital, age, gender, race, survival times, alive or dead), tumor information (primary site, tumor size, laterality, TNM stage, liver metastasis, and pulmonary metastasis), and pathology (histological type, pathological grade).

Exclusion criteria were listed below: (1) Age at the time of diagnosis younger than 18 years old; (2) Multiple primary tumors; (3) Incomplete data (missing demographic characteristics, tumor information, or survival data); (5) Autopsy cases; (6) Negative pathology report; (7) Incomplete information on lymph node metastasis.

International Classification of Diseases for Oncology code was employed for the histological subtype. The 7th TNM classification of the AJCC Cancer Staging Manual was used to determine oncology staging. The grade of pathological was defined as well-differentiated, moderately differentiated, poorly differentiated, undifferentiated, or unknown.

### Model construction and statistical analysis

The *t*-test and chi-square tests were used in this study. Screening variables to reduce overfitting of the multifactor models, the least absolute shrinkage and selection operator (Lasso) regression analysis was performed in the training cohort (28), then univariate analysis was applied to the variables with non-zero coefficients to further reduce irrelevant variables. Finally, we take variables that indicated statistical significance in univariate analysis into consideration in multivariate analysis, and the independent variables were identified to generate ML models. The final candidates for the ML models were identified by variables with P-value less than 0.05.

Models evaluated the probability of LNM for patients with RCC, based on six ML algorithms, including Logistic regression (LR). B.Naive Bayes Classifier (NBC).C. Decision tree (DT).D.Random Forest (RF). E.Gradient boosting machine (GBM). F.Extreme gradient boosting (XGB).These models were 10-fold cross-validated in the training cohort and validation cohort. The 10-fold cross-validation is to randomly divide the patients into training and validation sets in the ratio of 9:1, with 9 of them as the training set and 1 as the validation set each time. 10 times of validation are calculated. Each ML classifier was assessed *via* the receiver operating characteristic curve (ROC), the high area under ROC (AUC) represents high predictive accuracy (8). Assessing the weights of variables, permutation importance, and a heat map were employed to show the importance and correlation between the variables. Furthermore, the predictive performance was assessed by probability density function (PDF) and clinical utility curve (CUC) (29, 30).

Statistical analyses were performed in R software (version 4.0.5, https://www.r-project.org/). Python software (version3.8) was applied for developing an ML predictive model and web calculator. P <0.05 indicated positive statistical significance.

## Results

### Demographic and clinicopathological features

39016 patients with kidney cancer were enrolled in our study, LNM-related patients were 2544 cases (6.5%) in the this study cohort. M stage, marital,age, Sequence number,sex,primary site, grade,laterality, pathology, T stage and tumor size showed significant differences between N0 group and N1 group. The details was described in **Table 1**.

**Table 1:** Baseline of renal cell carcinoma patients with and without lymphatic metastasis.

Characteristics	level	N0 (N=36472)	N1 (N=2544)	p
M (%)	M0	33724 (92.47)	917 (36.05)	<0.0001
	M1	2748 (7.53)	1627 (63.95)	
Marital (%)	Married	22673 (62.17)	1470 (57.78)	<0.0001
	unmarried	13799 (37.83)	1074 (42.22)	
Age (median [IQR])	NA	64.000 [55.000, 72.000]	66.000 [57.000, 76.000]	<0.0001
Race.ethnicity (%)	black	4621 (12.67)	294 (11.56)	0.3699
	Chinese	445 (1.22)	29 (1.14)	
	other	2877 (7.89)	196 (7.70)	
	white	28529 (78.22)	2025 (79.60)	
Sequence number (%)	more	12318 (33.77)	607 (23.86)	<0.0001
	One primary only	24154 (66.23)	1937 (76.14)	
times (median [IQR])	NA	37.000 [14.000, 66.000]	8.000 [2.750, 19.000]	<0.0001
status (%)	alive	28338 (77.70)	616 (24.21)	<0.0001
	dead	8134 (22.30)	1928 (75.79)	
Sex (%)	female	12953 (35.51)	822 (32.31)	0.0012
	male	23519 (64.49)	1722 (67.69)	
Primary.Site (%)	C64.9-Kidney	34925 (95.76)	2166 (85.14)	<0.0001
	C65.9-Renal pelvis	1547 (4.24)	378 (14.86)	
Grade (%)	Moderately differentiated	13350 (36.60)	162 (6.37)	<0.0001
	Poorly differentiated	7711 (21.14)	573 (22.52)	
	Undifferentiated; anaplastic	2530 (6.94)	595 (23.39)	
	unknown	9810 (26.90)	1196 (47.01)	
	Well differentiated	3071 (8.42)	18 (0.71)	
Laterality (%)	left	17892 (49.06)	1399 (54.99)	<0.0001
	other	53 (0.15)	14 (0.55)	
	right	18527 (50.80)	1131 (44.46)	
Pathological (%)	8120/3:	720 (1.97)	311 (12.22)	<0.0001
	8130/3	877 (2.40)	86 (3.38)	
	8260/3	4665 (12.79)	225 (8.84)	
	8310/3	20031 (54.92)	772 (30.35)	
	8312/3	6270 (17.19)	717 (28.18)	
	8317/3	2035 (5.58)	39 (1.53)	
	other(n<1000)	1874 (5.14)	394 (15.49)	
T (%)	T1	25302 (69.37)	417 (16.39)	<0.0001
	T2	3532 (9.68)	371 (14.58)	
	T3	6634 (18.19)	1130 (44.42)	
	T4	580 (1.59)	444 (17.45)	
	TX	424 (1.16)	182 (7.15)	
Tumor Size (median [IQR])	NA	40.000 [25.000, 62.000]	80.000 [55.000, 111.250]	<0.0001

Pathological:8310/3: Clear cell adenocarcinoma, NOS, 8312/3: Renal cell carcinoma, 8260/3: Papillary adenocarcinoma, NOS,8317/3: Renal cell carcinoma, chromophobe type,8120/3: Transitional cell carcinoma, NOS,8130/3: Papillary transitional cell carcinoma, other (n<1000).

### Correlation of variable with clinical outcome

**Figure 1:**
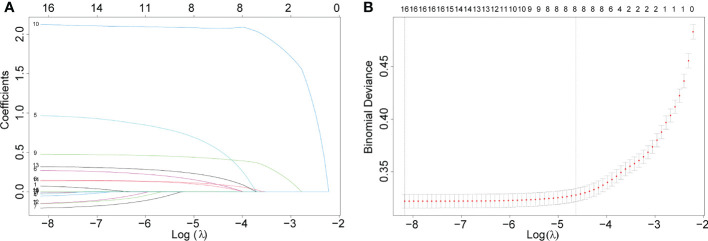
Eight variables were screened with nonzero coefficients when LNM was the endpoint. **(A)**. The results of the least absolute shrinkage and selection operator (LASSO) regression **(B)**.10-fold cross-validation of six machine learning algorithms.

LASSO regression was used to screen eight variables from sixteen variables with nonzero coefficients when LNM was the endpoint (**Figure 1**). Univariate analysis showed that age, grade, liver metastasis, M stage, primary site, pulmonary metastasis, T stage, and tumor size were associated with LNM. According to our multivariate logistic regression analysis findings, grade, liver metastasis, M stage, primary site, tumor size, and T stage were independent LNM risk factors. The age of patients does not show significant differences between LNM and non-LNM. Patients with a primary site in the C64.9-kidney had a higher risk of suffering LNM than patients with a primary site in the C65.9-renal pelvis. The patient will face a greater danger of occurrence of LNM when the pathological level turns bad, except moderately differentiated. Liver metastasis was identified to be an independent risk factor, but pulmonary metastasis could not be a risk factor. Moreover, patients with higher M stage (M1) and T stage (T1, T2, T3, T4) were accompanied by more dangers. The detailed data was demonstrated in **Table 2**.

**Table 2:** Univariate and multivariate logistic regression analysis of risk factors for lymph node metastasis in patients with renal cell cancer.

Characteristics	Univariate logistic			Multivariable logistics		
	OR	CI	*P*	OR	CI	*P*
Age	1.01	1.01-1.02	<0.001	1.00	0.99-1.00	0.134
Grade						
Well-differentiated	Ref	Ref	Ref	Ref	Ref	Ref
Moderately differentiated	2.07	1.27-3.37	0.003	1.46	0.89-2.4	0.136
Poorly differentiated	12.68	7.92-20.3	<0.001	4.35	2.69-7.04	<0.001
Undifferentiated; anaplastic	40.12	25.03-64.31	<0.001	5.36	3.29-8.73	<0.001
unknown	20.8	13.04-33.18	<0.001	5.54	3.44-8.95	<0.001
Liver. metastasis						
No	Ref	Ref	Ref	Ref	Ref	Ref
Yes	16.32	14.2-18.77	<0.001	1.59	1.35-1.87	<0.001
Unknown	10.52	5.93-18.65	<0.001	1.17	0.62-2.2	0.622
M						
M0	Ref	Ref	Ref	Ref	Ref	Ref
M1	21.77	19.9-23.82	<0.001	7.92	6.88-9.11	<0.001
Primary.Site						
C64.9-Kidney	Ref	Ref	Ref	Ref	Ref	Ref
C65.9-Renal pelvis	3.94	3.49-4.44	<0.001	3.33	2.83-3.92	<0.001
Pulmonary. metastasis						
No	Ref	Ref	Ref	Ref	Ref	Ref
Yes	14.96	13.63-16.42	<0.001	1.12	0.98-1.28	0.108
T						
T1	Ref	Ref	Ref	Ref	Ref	Ref
T2	6.37	5.52-7.36	<0.001	2.48	2.09-2.93	<0.001
T3	10.34	9.21-11.6	<0.001	3.73	3.25-4.29	<0.001
T4	46.45	39.7-54.34	<0.001	6.89	5.68-8.35	<0.001
TX	26.04	21.35-31.77	<0.001	3.75	2.98-4.73	<0.001
Tumor.Size	1.02	1.02-1.02	<0.001	1.00	1.00-1.00	<0.001

### Development and validation of predictive models

**Figure 2:**
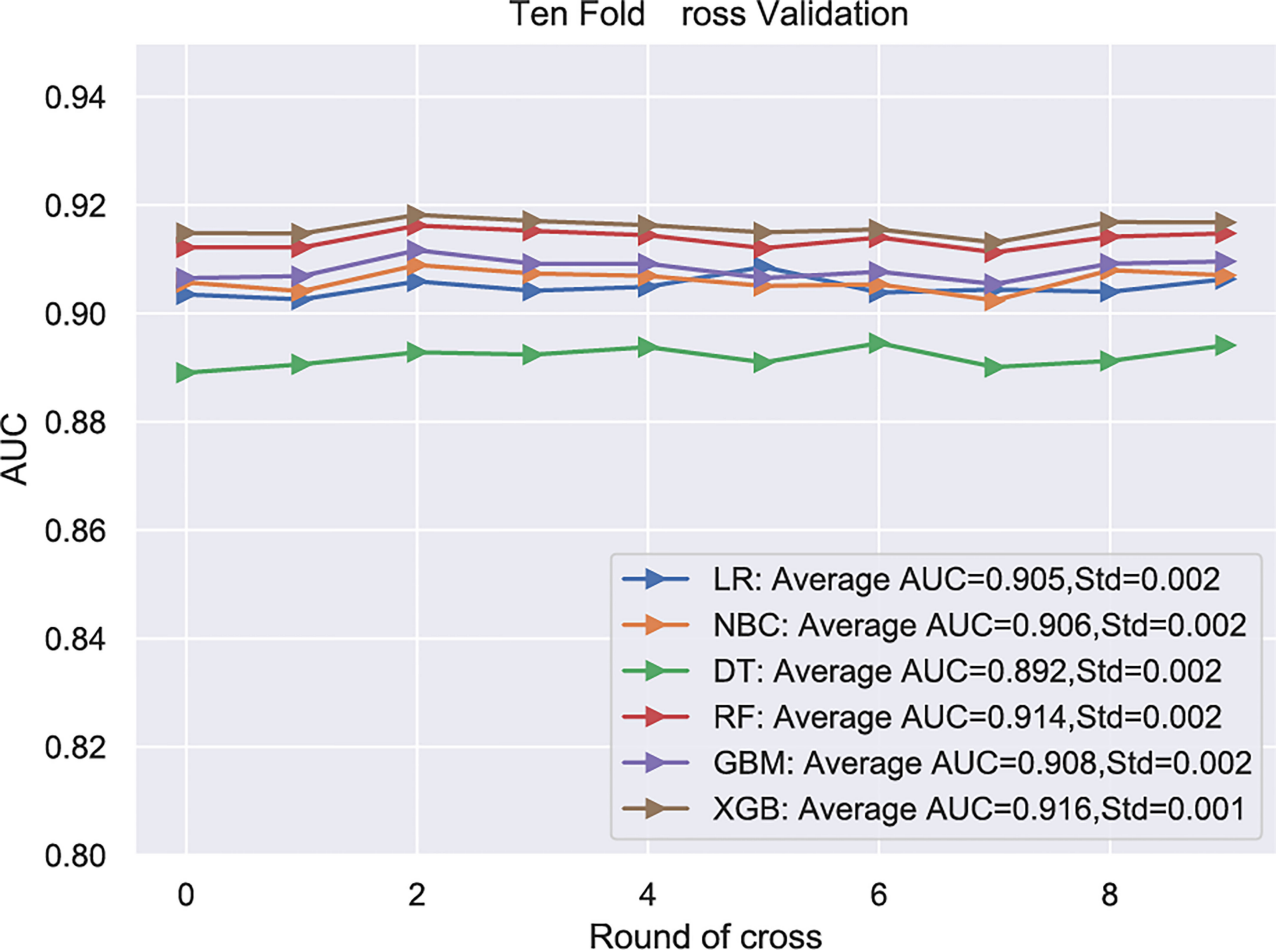
.Among all ML-ed models, the XGB model showed the best predictive performance. LR, Logistic regression; NBC, Naive Bayes Classifier; DT, Decision tree; RF, Random Forest; GBM, Gradient boosting machine; XGB, Extreme gradient boosting; Std, Standard Deviation.

**Figure 3:**
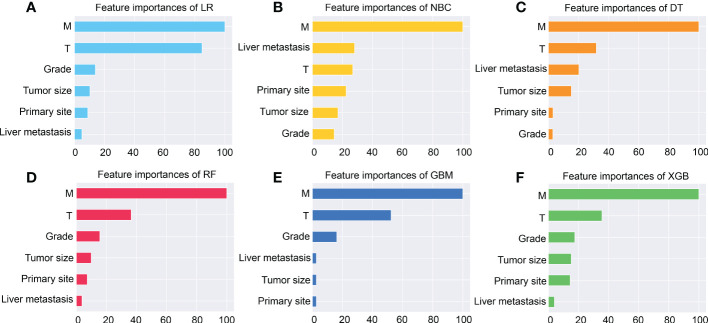
The importance of Variables in each prediction model. Among these factors, the M stage is the most important one. **(A)** Logistic regression (LR). **(B)** Naive Bayes Classifier (NBC). **(C)** Decision tree (DT). **(D)** Random Forest (RF). **(E)** Gradient boosting machine (GBM). **(F)** Extreme gradient boosting (XGB).

**Figure 4:**
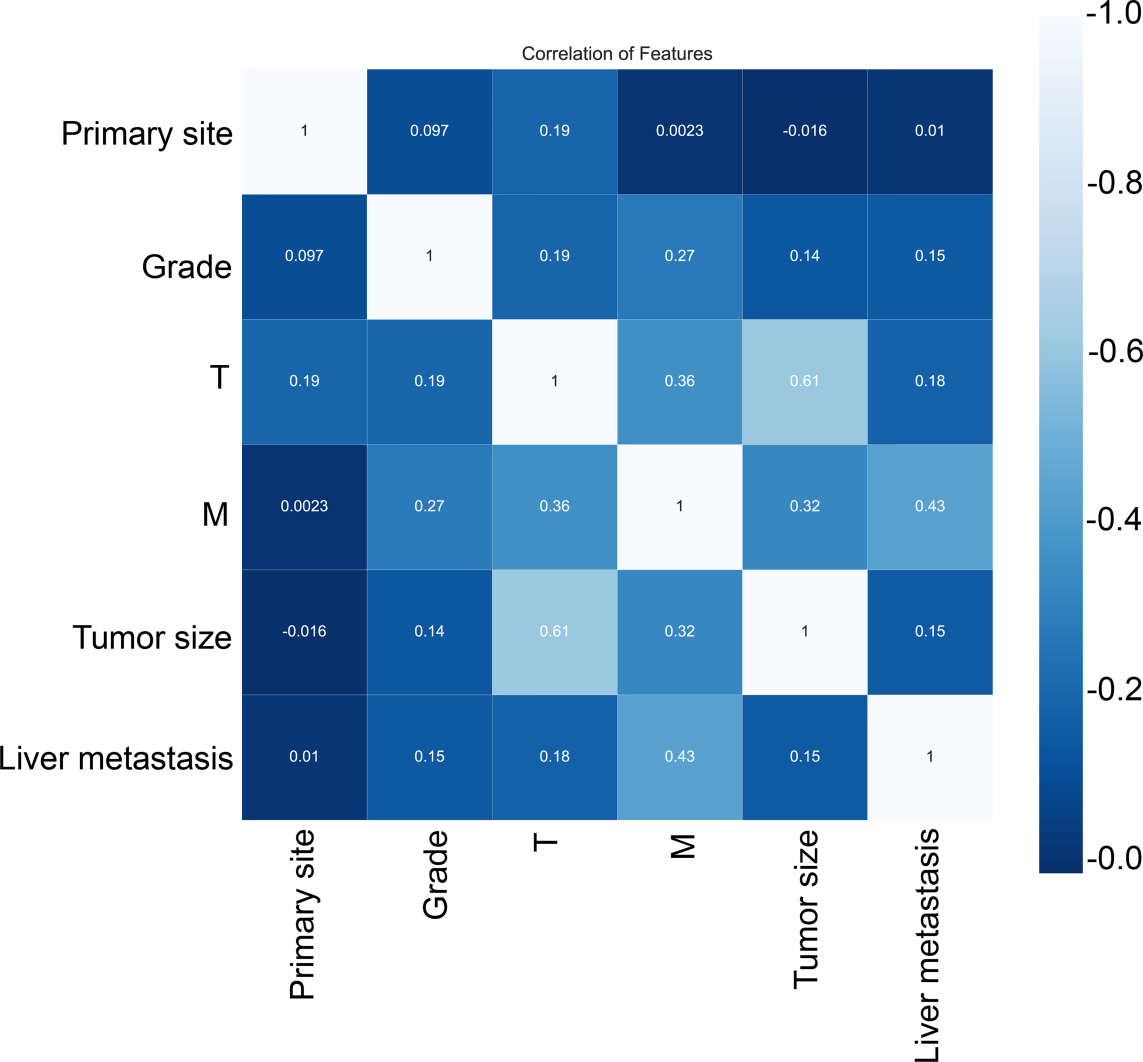
Correlation of variables. These variables were independent of each other with no significant correlation and no collinearity.

Subsequently, multivariate analysis results yielded six independent risk factors which constituted the basis for our ML models. According to the results of the 10-fold cross-validation in the training cohort, the average AUC values of six ML-based models were listed in **Figure 2**. Among all ML-ed models, the XGB model showed the best predictive performance (AUC = 0.916, SD = 0.001), closely followed by RF (AUC = 0.914, SD = 0.002), GBM (AUC = 0.908, SD = 0.002) and NBC (AUC = 0.906, SD = 0.002), while the performance of DT (AUC = 0.892, SD = 0.002) was poor. As for the conventional method, LR also performed well (AUC = 0.905, SD = 0.002) (**Figure 2**). Consequently, the XGB model was used as the optimal prediction model. **Figure 3** showed the relative importance of six variables in each prediction model and common trends among all algorithms: the M stage ranked first in all variables. In the XGB model, M stage, T stage, and pathological grade were the top three important variables. We evaluated the correlation of the variables in **Figure 4** with a heat map. There was no significant correlation and no collinearity, and variables were independent of each other.

### Choice of the best threshold probability

**Figure 5:**
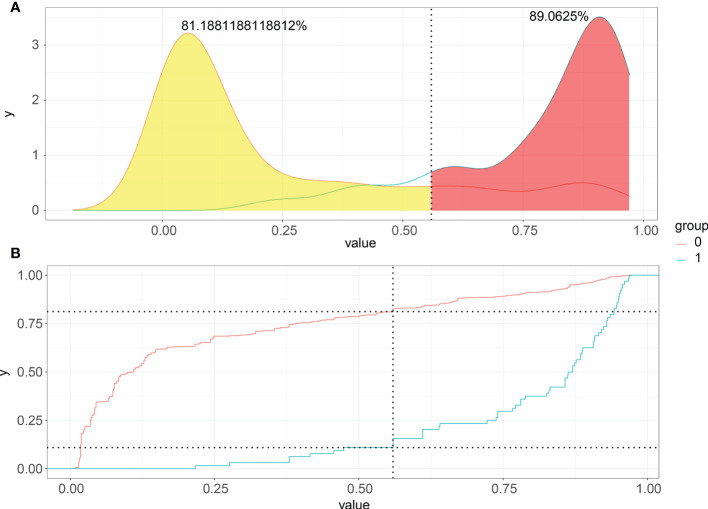
Probability density functions and Clinical utility curves of the predictive model. **(A)** Non-LNM patients were mostly concentrated in the portion representing 0-54.6% LNM risk, and LNM were distributed in the residual section; **(B)** The true positive percentage of LNM and non-LNM at any threshold of probabilities.

Choosing the better threshold and clinical performance of the XGB model, PDF, and CUC results could be the decisive factor. Although a moderate overlap between the two curves in PDF, we can see that non-LNM patients were mostly concentrated in the portion representing 0-54.6% LNM risk, whereas patients with LNM were distributed in the residual section (**Figure 5A**). The CUC presented the true positive percentage of LNM and non-LNM at any threshold of probabilities (**Figure 5B**). In clinical practice, correct detection of LNM has the same importance as the diagnosis of no LNM. In our study, 54.6% was chosen as the threshold probability for making a clinical decision. About 81% of non-LNM patients and about 89% of LNM patients could be determined.

### Risk prediction of lymph node metastasis in patients with renal cell carcinoma

**Figure 6:**
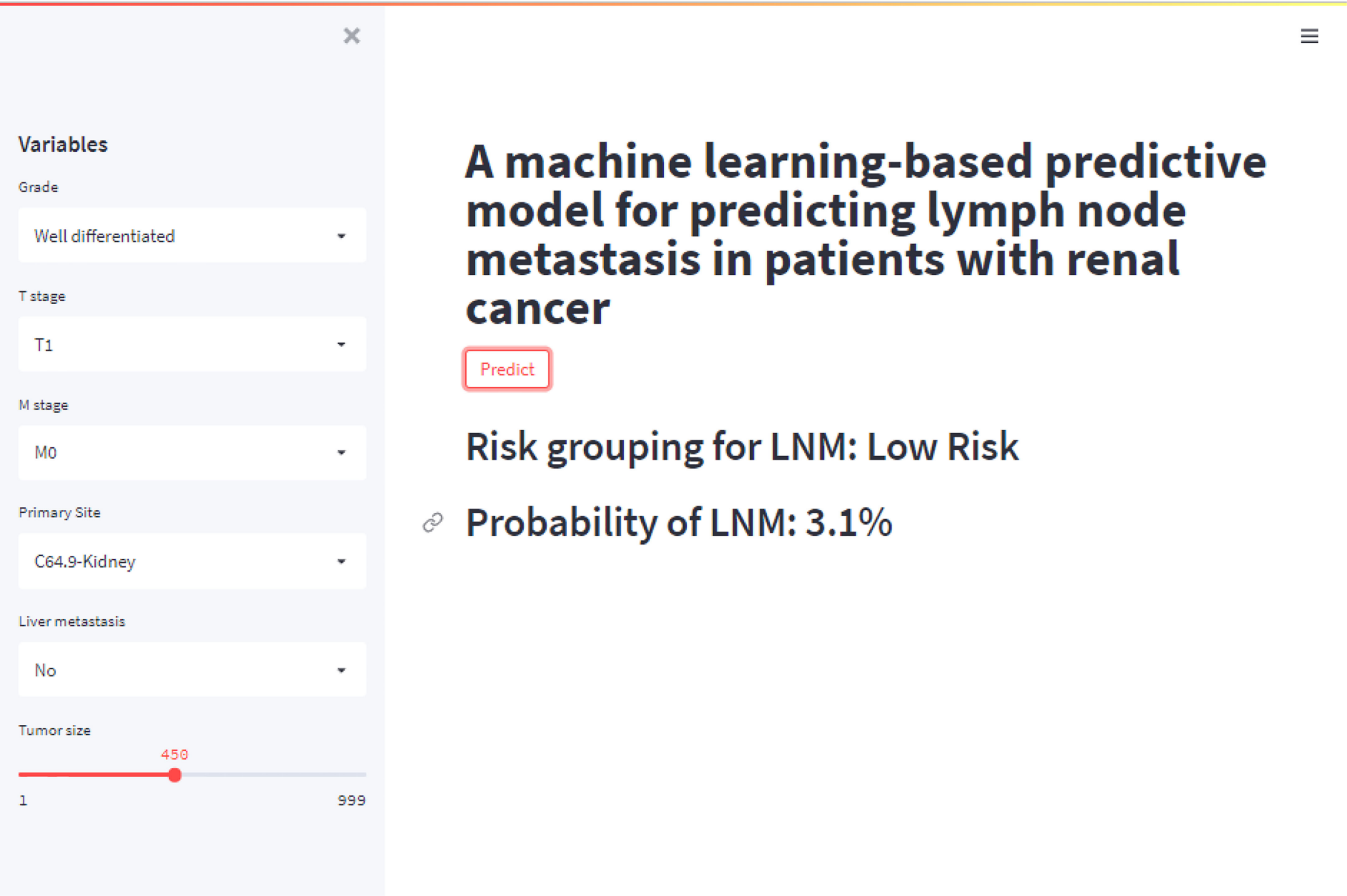
The web calculator for predicting lymph node metastasis in patients with RCC.

A web calculator were built based on the XGB model with six variables for clinicians to predict patients’ corresponding probability of LNM by the input of variables (The concise tool can be acquired by clicking on the link below: https://share.streamlit.io/liuwencai4/renal_lnm/main/renal_lnm.py). As shown in **Figure 6**, we calculated the probability online quickly (Probability of LNM = 3.1%).

## Discussion

Kidney cancer is the third most frequent cancer in the urological tumor (31). Approximately one-third of patients with kidney cancer will develop metastases, of whom 2.7 to 10% lived with lymph node metastasis (11). Lymph node metastasis harms the prognosis of kidney cancer (11, 12, 32–34). 3-years of OS rates of patients with or without LNM were 86.5% and 61.1% respectively (35). Unfortunately, we still do not have effective systemic treatment for metastatic kidney cancer (1). Implementing early diagnosis can assist clinicians in decision-making in this dilemma situation.

In the present study, we validated six ML models to predict LNM in RCC. There were five significant findings. First, the six independent risk factors of LNM were pathological grade, liver metastasis, M stage, primary site, T stage, and tumor size. Second, the six ML models could predict LNM, most models reached high areas under the ROC curve (AUCs) > 0.9. Third, after comparing the performance of the six ML-based models, XGB had the best prediction performance. Fourth, 54.6% of the threshold probability for clinical decision-making was identified by FDP and CUC. Fifth, we built a web calculator based on the XGB model.

In our study, T stage, M stage, and pathological grade were independent predictors of LNM, Patients with high-level pathological grade, and advanced T and M stages had a higher risk of LNM, probably indicating that tumors were closely related to much more drastic aggressive, which was similar to previous studies (17, 18, 36–38). Meanwhile, **Figure 5** also showed that the T stage and M stage were the top two important variables in our five models. Noticeably, the M stage had a powerful impact on LNM, surpassing that of the T stage. T and M stages were correlated with poor survival of RCC patients (7). Therefore, T and M stages play a crucial role in the diagnosis, clinical management, and prognosis. We suggested a whole-body CT scan, even PET/CT is necessary for identifying LNM. Furthermore, we also found a tight correlation between tumor size and the incidence of LNM in RCC, and this correlation has been revealed previously (17, 39, 40). Thompson et al. demonstrated that tumor size was closely associated with poor prognosis and RCC patients with a tumor smaller than 3 cm had a significantly low risk of metastasis (39). Nevertheless, Kates et al. showed that patients with tumors 25 ~ 30 mm in size still had a greater metastatic potential (40). Liver metastasis increased the 1.59-fold risk in LNM patients compared with patients without. Although lung metastasis was a risk factor for LNM based on the result of the univariate analysis(*p* < 0.001), multivariate analysis didn’t yield a significant difference. According to the origin, RCC could be subdivided into renal pelvic RCC and kidney cancer. In our study, these two subtypes had a distinct difference in LNM possibility. The risk of renal pelvic LNM was 3.33 times higher than kidney cancer. Renal pelvis RCC with an invasion of the inferior vena cava or the renal vein may induce early-onset metastasis (41). Hematogenous spread of tumor cells may increase the occurrence of LNM. However, a study, which enrolled 2485 patients with RCC, demonstrated that the location of primary RCC tumors does not increase the risk of LNM (42).

Multivariate prediction tools were established based on hematological and serum biochemical variables, radiological features, and pathological and molecular parameters (17, 18, 32, 42, 43). We first develop and validate predictive models with ML algorithms. eligible patients from the SEER database were selected for our model development cohort, our sample size was the largest. Six ML algorithms had well performance in predicting LNM. Comparing the performance in internal validation with 10-fold cross-validation, we found that the XGB algorithm was better than those of other algorithms (AUC = 0.916). Furthermore, the XGB still had the best performance by externally validating in a cohort from a Chinese medical institution (AUC=0.915).

ML technology has been widely applied in the healthcare field with powerful computing capacity. This AI technology could predict the possibility of metastatic diseases, aid diagnosis, and evaluate prognosis by analyzing, training, and modeling a bulk of medical data within a short period (44). Predicting lymph node metastasis with ML algorithms has been proven in lung, thyroid, and colorectal cancer (22, 24, 25, 45–47). The XGB method was an ensemble learning method (48). This ML algorithm can minimize errors, maximize models’ performance, and effectively prevents overfitting (44, 49).

Probability density function (PDF) and Clinical utility curve (CUC) were used to compare the net benefit of different thresholds in our study. 54.6% of the threshold probability was chosen as the best cut-off value for clinical decision-making. Patients with a higher metastatic risk than 54.6% were classified as a high-risk group of LNM. In our ML-based model, approximately 89% of metastatic patients can be detected. To improve the availability and clinical usefulness, we set up a web calculator of the XGB model with six variables introduced. The web calculator of the RFC model can provide a visual and dynamic assessment. By typing the patient’s personalized information into this web calculator, clinicians and patients from anywhere in the world can easily obtain the probability of LNM and evaluate the LNM risk.This model could easily process the association between the predictors and LNM and it could be a useful method for other patients worldwide.

In addition to the clinical findings, innovations in the present study are listed below. First, this is the first study to develop ML-based models for the prediction of LNM in RCC patients. Furthermore, the accuracy and reliability of the ML-based models have been verified by an external validation cohort. External validation was executed to verify the accuracy and reliability. Additionally, we applied permutation importance to identify the importance of each variable and used a correlation heat map to explore the correlation between variables. Finally, we established an online application of the XGB to calculate the risk for each patient.

Our study still has several limitations. First, the data from external validation and the SEER database were retrospectively selected. This could lead to selection bias, a prospective cohort could be designed to enhance the credibility of the results in the future. Secondly, despite including a large sample size and achieving high accuracy, several candidate variables which have been previously explored were not involved in this study, such as the presence of a sarcomatous component, ECOG-PS, histological tumor necrosis, clinical node status, local symptoms, molecular and gen parameters, the lactate dehydrogenase level, and the Fuhrman classification. These variables could improve the accuracy of the model prediction. Meanwhile, we extracted the patients according to ICD-O codes, not the latest published WHO histological types.

## Conclusions

In our study, we innovatively comprehensively assess several ML-based predictive models and reported the XGB algorithm could be the optimal model for predicting LNM in RCC patients. Six independent risk factors of LNM were identified, including grade, liver metastasis, M stage, primary site, T stage, and tumor size. Considering that patients with genotypically or phenotypically different, RCC may correspond to a different association between the predictors and LNM. In addition to the developed cohort origin from the SEER database, this model also has been externally validated in regions of China. The PDF and CUC showed that our tool works well in clinical utility. The web calculator of the RFC model can provide a visual and dynamic assessment. This model could easily process the association between the predictors and LNM in our database and it could be a useful method for other patients worldwide.

## Data availability statement

The original contributions presented in the study are included in the article/supplementary material. Further inquiries can be directed to the corresponding authors.

## Ethics statement

Ethical review and approval was not required for the study on human participants in accordance with the local legislation and institutional requirements. Written informed consent from the participants’ legal guardian/next of kin was not required to participate in this study in accordance with the national legislation and the institutional requirements.

## Author contributions

Conceptualization, CLY, HZ and WLL. Data curation, XWF, TH and WCL. Formal analysis, XWF, TH and WCL. Investigation, CLY and CX.Methodology, WLL and WYL. Resources, BY and YS. Validation,TL. Writing – original draft, XWF. Writing – review & editing, WLL and CLY. All authors contributed to the article and approved the submitted version.

## Funding

This research was funded by the Science Foundation of The Tianjin Education Commission, grant number 2018KJ075.

## Conflict of interest

BY was employed by Tianjin Prosel Biological Technology Co., Ltd.

The remaining authors declare that the research was conducted in the absence of any commercial or financial relationships that could be construed as a potential conflict of interest.

## Publisher’s note

All claims expressed in this article are solely those of the authors and do not necessarily represent those of their affiliated organizations, or those of the publisher, the editors and the reviewers. Any product that may be evaluated in this article, or claim that may be made by its manufacturer, is not guaranteed or endorsed by the publisher.
